# Epidemiology of Bleeding in Critically Ill Children

**DOI:** 10.3389/fped.2021.699991

**Published:** 2021-08-04

**Authors:** Jake Sequeira, Marianne E. Nellis, Oliver Karam

**Affiliations:** ^1^Division of Pediatric Critical Care Medicine, Children's Hospital of Richmond at Virginia Commonwealth University, Richmond, VA, United States; ^2^Pediatric Critical Care Medicine, NY Presbyterian Hospital – Weill Cornell Medicine, New York, NY, United States

**Keywords:** critical illness, hemostasis, intensive care units, pediatric, hemorrhage, demographic, child

## Abstract

**Objective:** Bleeding can be a severe complication of critical illness, but its true epidemiologic impact on children has seldom been studied. Our objective is to describe the epidemiology of bleeding in critically ill children, using a validated clinical tool, as well as the hemostatic interventions and clinical outcomes associated with bleeding.

**Design:** Prospective observational cohort study.

**Setting:** Tertiary pediatric critical care unit

**Patients:** All consecutive patients (1 month to 18 years of age) admitted to a tertiary pediatric critical care unit

**Measurements and Main Results:** Bleeding events were categorized as minimal, moderate, severe, or fatal, according to the Bleeding Assessment Scale in Critically Ill Children. We collected demographics and severity at admission, as evaluated by the Pediatric Index of Mortality. We used regression models to compare the severity of bleeding with outcomes adjusting for age, surgery, and severity. Over 12 months, 902 critically ill patients were enrolled. The median age was 64 months (IQR 17; 159), the median admission predicted risk of mortality was 0.5% (IQR 0.2; 1.4), and 24% were post-surgical. Eighteen percent of patients experienced at least one bleeding event. The highest severity of bleeding was minimal for 7.9% of patients, moderate for 5.8%, severe for 3.8%, and fatal for 0.1%. Adjusting for age, severity at admission, medical diagnosis, type of surgery, and duration of surgery, bleeding severity was independently associated with fewer ventilator-free days (*p* < 0.001) and fewer PICU-free days (*p* < 0.001). Adjusting for the same variables, bleeding severity was independently associated with an increased risk of mortality (adjusted odds ratio for each bleeding category 2.4, 95% CI 1.5; 3.7, *p* < 0.001).

**Conclusion:** Our data indicate bleeding occurs in nearly one-fifth of all critically ill children, and that higher severity of bleeding was independently associated with worse clinical outcome. Further multicenter studies are required to better understand the impact of bleeding in critically ill children.

## Introduction

Bleeding is a severe complication of critical illness in children, which has been associated with longer hospital stays, increased blood product and vasoactive utilization, as well as increased mortality (1, 2). Despite the burden bleeding places on the critically ill pediatric community, to date there remains little clarity regarding its incidence, with wide variations recorded in pediatric populations of different ages (2, 3). Additionally, adult studies make use of different clinical definitions of bleeding, which when coupled with the intrinsic differences in incidence and physiology of disease states in the adult population, make generalization to the pediatric population difficult (4). This lack of pediatric epidemiological data presents a major obstacle in designing studies that evaluate interventions to prevent or stop bleeding.

Patient blood management programs have effectively reduced incidence of transfusions, with restrictive transfusion strategies being employed in part because of a better understanding of the physiologic impact of bleeding (5–8). It is not known if restrictive transfusion strategies in bleeding adult patients can be extrapolated to children, in part because of the challenge of an objective description of bleeding and lack of epidemiological background (8). Our objective in this study was to describe the epidemiology of bleeding in the critically ill population, using a clinical tool validated specifically for use in critically ill children (9).

## Methods

We conducted a prospective longitudinal observational study in a tertiary 20-bed pediatric intensive care unit (PICU) in Richmond, Virginia. From November 2019 to October 2020, we enrolled all consecutive critically ill children (1 month to 18 years of age) admitted to the PICU. We excluded preterm infants (<44 weeks gestation at age of enrollment) or term infants <28 days old, as bleeding etiologies are specific to the neonatal period and the bleeding assessment tool is not calibrated for this population. In addition, we excluded patients with known limitations of care at enrollment. The study was approved by the Institutional Review Board (HM20016435) with a waiver of individual consent.

Due to logistical constraints, clinical data was only collected Monday through Friday. For all critically ill children, we collected demographics (such as reason for admission, age, weight, surgical status), severity at admission as evaluated by the Pediatric Index of Mortality, length of mechanical ventilation and 28-day ventilator-free days, PICU length of stay and 28-day PICU-free days, and 28-day mortality (10). To avoid potential biases due to the unavailability of floor beds delaying transfers of children no longer critically ill, PICU discharge was based on two consecutive Pediatric Early Warning Score (PEWS) <3 (11).

### Outcome Variables

We separated our outcome variables into three categories: (1) epidemiology of bleeding severity; (2) risk factors for bleeding severity; and (3) association with clinical outcomes.

The epidemiology of the bleeding severity was evaluated with daily bleeding scoring, according to the Bleeding Assessment in Critical Illness (BASIC) definition (9). Bleeding assessments were made by a single observer, evaluating patients over the previous 24 h using chart review and nursing assessments. According to the definition, the severity of bleeding was based on progression to organ failure, hemodynamic instability, drop in hemoglobin (Hb), amount of quantifiable bleeding (e.g., chest tube), and some specific locations (intra-spinal, intra-articular, and intraocular bleeding).

Risk factors for bleeding severity were evaluated by comparing various demography variables, such as reason for admission, age, weight, severity at admission, surgical status, length of surgery, and type of surgery.

The association with outcomes were evaluated by the length of mechanical ventilation and 28-day ventilator-free days, PICU length of stay and 28-day PICU-free days, and 28-day mortality. For patients who experienced bleeding, we collected interventions to treat bleeding including transfuSsion of blood products like packed red cells, platelets, cryoprecipitate, and fresh frozen plasma. We also collected use of other hemostatic products (antifibrinolytics, prothrombin complex concentrates, rFVIIa, or other factor concentrates), decreasing anticoagulation, and surgical procedures to treat bleeding (surgery to maintain hemostasis, chest tube drainage, etc.,).

### Sample Size

Sample size was based on precision of ± 5% around the proportion of severe bleeding. Based on pediatric and adult preliminary data, we estimated 45% of critically ill children would have at least one episode of bleeding during their PICU stay, 20% of which would be severe bleeding (2, 4). Therefore, to obtain the 95% confidence interval of a 20% proportion of severe bleeding, between 15 and 25%, we would need to include 250 bleeding critically ill children. Anticipating that 55% of the patients would not bleed, we anticipated enrolling 305 (55%) non-bleeding patients, for a total of 555 patients. We had decided a priori to continue the enrollment up to a year if the number of bleeding patients was lower than 250.

### Statistical Analysis

Descriptive statistics are reported as mean ± SD, median and interquartile range (IQR), or proportions with their 95% confidence interval (95% CI).

We compared demographic variables and outcomes between the various levels of severity of bleeding (no bleeding, minimal bleeding, moderate bleeding, and severe bleeding) with a Fisher exact test (for proportions) and Kruskal-Wallis (for continuous variables). For these comparisons, the only case of fatal bleeding was combined with severe bleeding. We compared the interventions to stop bleeding similarly.

Multivariable models were developed to evaluate the severity of bleeding, ventilator-free days, PICU-free days, and 28-day mortality. We used logistic regression models for binary outcomes (mortality), ordinal regressions for ordinal outcomes (severity of bleeding), and linear regression models for continuous outcomes (ventilator-free days, PICU-free days). Variables were considered potential predictors if they were associated with the outcome in univariate analysis (*p*< 0.10). The final model was selected using a bidirectional step-wise selection on the potential predictors with a significance criterion of the *p*-value of <0.05 to enter in the model and a *p*-value of >0.1 to be removed. As age and weight were collinear, we only included age in the model. No variables other than the severity of bleeding were forced into the models.

All tests were two-sided, with an alpha level of 0.05. All statistical analyses were performed with SPSS version 27 for the Mac (SPSS, Chicago, IL).

## Results

### Demographics

As seen in Table 1, over 52 weeks, 902 critically ill patients were enrolled, inclusive of readmissions. The median age was 64 months (IQR 17; 159), the median admission predicted risk of mortality was 0.5% (IQR 0.2; 1.4); and 24% of the children were post-surgical. The most common overall admission diagnoses were respiratory distress, elective surgery, and sepsis, with elective surgery representing 32% of all bleeding patients. One patient required extracorporeal life support (ECMO).

**Table 1:** Demographics (*n* = 902).

**Variables**	**Highest severity of bleeding**				***P*-value**
	**No bleeding (*n* = 744)**	**Minimal bleeding (*n* = 71)**	**Moderate bleeding (*n* = 52)**	**Severe bleeding** ^*^ **(*n* = 35)**	
Male	406 (54.6%)	43 (60.6%)	32 (61.5%)	22 (62.9)	0.49
Age [months]	59 (16; 149)	121 (24; 184)	102 (12; 174)	102 (36; 170)	0.02
Weight [kg]	18.4 (10.4; 43.9)	29.7 (13.8; 54.3)	22.1 (8.4; 48.1)	25.0 (12.2; 41.0)	0.05
PIM-2 risk of mortality [%]	0.4 (0.2; 1.1)	0.5 (0.2; 5.1)	1.2 (0.4; 97.2)	6.8 (0.8; 99.9)	<0.001
Primary diagnosis					<0.001
Hypovolemic shock	3 (0.4%)	0 (0%)	0 (0%)	1 (2.9%)	
Severe sepsis and septic shock	23 (3.1%)	7 (9.9%)	1 (1.9%)	0 (0%)	
Hemorrhagic shock	0 (0%)	0 (0%)	0 (0%)	1 (2.9%)	
Cardiogenic shock	5 (0.7%)	1 (1.4%)	0 (0%)	0 (0%)	
Burn	11 (1.5%)	2 (2.8%)	0 (0%)	0 (0%)	
Traumatic brain injury	8 (1.1%)	1 (1.4%)	2 (3.8%)	1 (2.9%)	
Trauma	50 (6.7%)	4 (5.6%)	7 (13.5%)	6 (17.1%)	
Cardiac surgery (bypass)	0 (0%)	1 (1.4%)	10 (19.2%)	2 (5.7%)	
Cardiac surgery (no bypass)	2 (0.3%)	0 (0%)	0 (0%)	0 (0%)	
Cardiac procedure (non-surgical)	9 (1.2%)	0 (0%)	0 (0%)	0 (0%)	
Emergency surgery	13 (1.7%)	3 (4.2%)	2 (3.8%)	5 (14.3%)	
Elective surgery	106 (14.2%)	27 (38%)	16 (30.8%)	8 (22.9%)	
Seizures	50 (6.7%)	2 (2.8%)	0 (0%)	1 (2.9%)	
Encephalopathy	21 (2.8%)	4 (5.6%)	1 (1.9%)	2 (5.7%)	
Renal failure	6 (0.8%)	0 (0%)	1 (1.9%)	0 (0%)	
Hepatic failure	1 (0.1%)	0 (0%)	0 (0%)	0 (0%)	
Respiratory distress (infectious)	140 (18.8%)	5 (7%)	1 (1.9%)	0 (0%)	
Respiratory distress (non-infectious)	96 (12.9%)	4 (5.6%)	3 (5.8%)	0 (0%)	
Oncology	48 (6.5%)	4 (5.6%)	6 (11.5%)	5 (14.3%)	
Endocrine (including DKA)	69 (9.3%)	0 (0%)	0 (0%)	0 (0%)	
Infection	18 (2.4%)	1 (1.4%)	0 (0%)	1 (2.9%)	
Neuro (other)	38 (5.1%)	2 (2.8%)	0 (0%)	0 (0%)	
Cardiac (medical)	3 (0.4%)	0 (0%)	0 (0%)	0 (0%)	
Other	24 (3.2%)	3 (4.2%)	2 (3.8%)	2 (5.7%)	
Surgical patients	132 (17.7%)	35 (49.3%)	31 (59.6%)	18 (51.4%)	<0.001
Type of surgeries					<0.001
Spinal	12 (9.1%)	0 (0%)	1 (3.2%)	0 (0%)	
Intracranial	49 (37.1%)	6 (17.1%)	3 (9.7%)	5 (27.8%)	
Cardiac	2 (1.5%)	1 (2.9%)	10 (32.3%)	2 (11.1%)	
Thoracic	6 (4.5%)	14 (40%)	2 (6.5%)	1 (5.6%)	
Orthopedic	4 (3%)	1 (2.9%)	3 (9.7%)	2 (11.1%)	
Cranial vault	10 (7.6%)	1 (2.9%)	0 (0%)	1 (5.6%)	
ENT	14 (10.6%)	0 (0%)	4 (12.9%)	1 (5.6%)	
Trauma	3 (2.3%)	4 (11.4%)	4 (12.9%)	2 (11.1%)	
Transplant	4 (3%)	2 (5.7%)	1 (3.2%)	1 (5.6%)	
Plastics	10 (7.6%)	1 (2.9%)	2 (6.5%)	0 (0%)	
Abdominal	8 (6.1%)	2 (5.7%)	0 (0%)	1 (5.6%)	
Renal and urology	4 (3%)	1 (2.9%)	0 (0%)	1 (5.6%)	
Other	6 (4.5%)	2 (5.7%)	1 (3.2%)	1 (5.6%)	
Length of surgery [hr]	2.5 (1.5; 4.0)	2.0 (1.0; 3.5)	4.0 (2.5; 6.0)	3.0 (.4; 7.8)	0.003
Surgery requiring vascular repair	12 (5.8%)	2 (4.9%)	10 (26.3%)	6 (22.2%)	<0.001
Surgery requiring hemostatic transfusions	25 (12.1%)	4 (9.5%)	17 (44.7%)	14 (51.8%)	<0.001
TXA in OR [mg/kg]	31 (26; 38)	18 (*n* = 3)	15 (14; 34)	30 (16; 94)	0.34
Platelets in OR [ml/kg]	4.6 (0.0; 8.0)	(*n* = 0)	5.9 (*n* = 3)	10.9 (5.2; 23.0)	0.21
Plasma in OR [ml/kg]	5.0 (0.0; 15.9)	(*n* = 0)	15.8 (9.1; 32.5)	18.9 (13.0; 43.5)	0.11
Cryo in OR [ml/kg]	0.0 (0.0; 0.0)	(*n* = 0)	(*n* = 0)	2.7 (*n* = 3)	0.11

* *Including 1 case of fatal bleeding (intracranial hemorrhage)*.

*For cells with ≤ 3 subjects, only medians are provided (not interquartile range). p-values represent the measure of the probability that an observed difference could have occurred just by random chance, across the four groups (no bleeding, minimal, moderate, and severe bleeding). For example, despite numerical differences in the weight, the differences in proportions are not statistically significant. On the other hand, the proportion of surgical patients is statistically significant, indicating that the distribution across the four groups is not due to chance (<0.1% of chance). PIM-2, pediatric index of mortality; DKA, diabetic ketoacidosis; TXA, tranexamic acid; OR, operating room*.

### Bleeding Events

**Figure 1:**
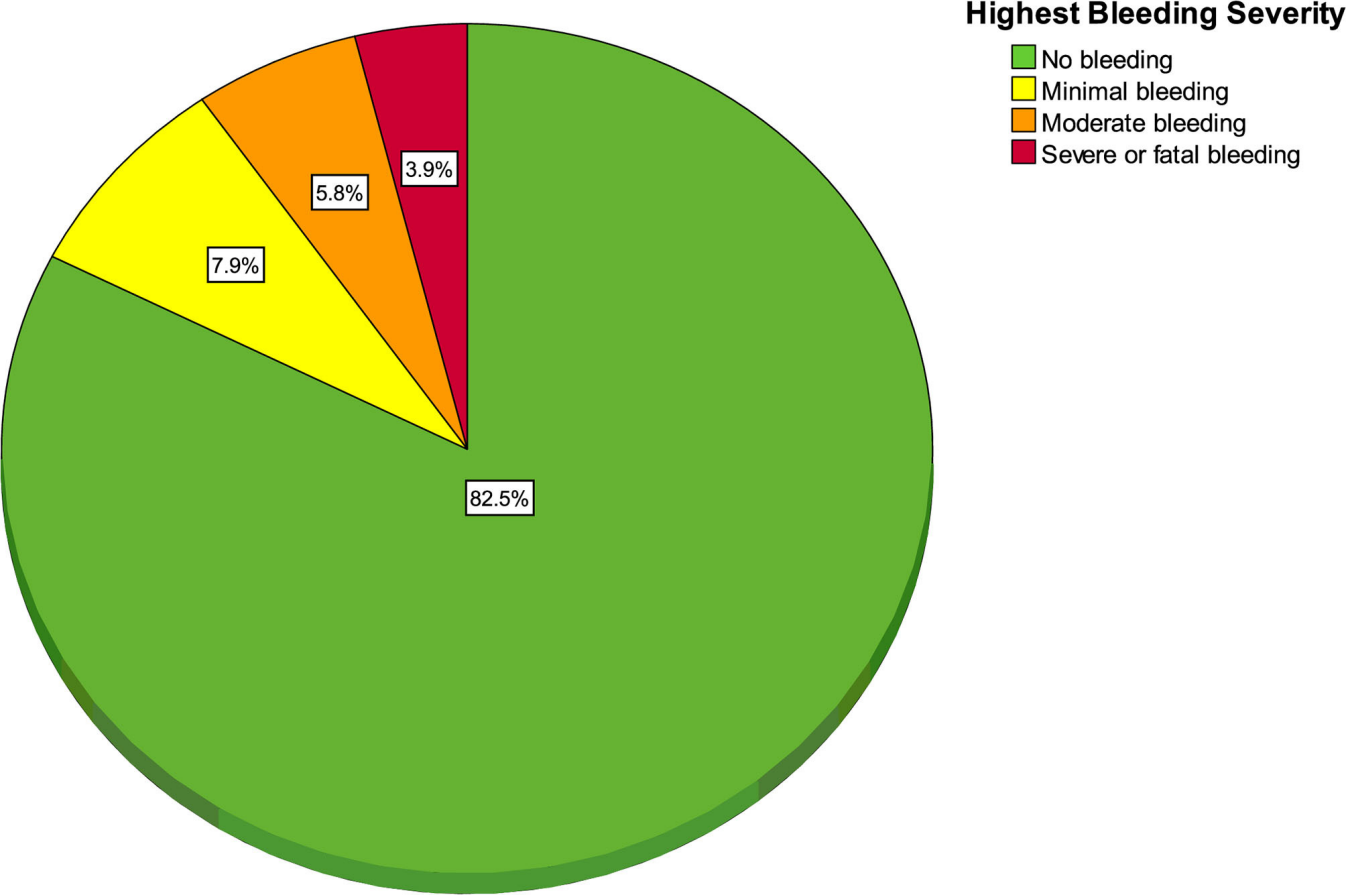
Pie chart of highest bleeding severity (*n* = 158 bleeding patients and 744 non-bleeding patients).

One hundred and fifty-eight patients (17.5%, 95% CI 15.0 to 20.0) experienced at least one bleeding event. As seen in Figure 1, the most frequent severity of bleeding was minimal for 71 patients (7.9%, 95% CI 6.3 to 9.8), moderate for 52 (5.8%, 95% CI 4.4 to 7.5), severe for 34 (3.8%, 95% CI 2.7 to 5.2) and fatal for 1 (0.1%, 95% CI 0 to 0.6%).

**Figure 2:**
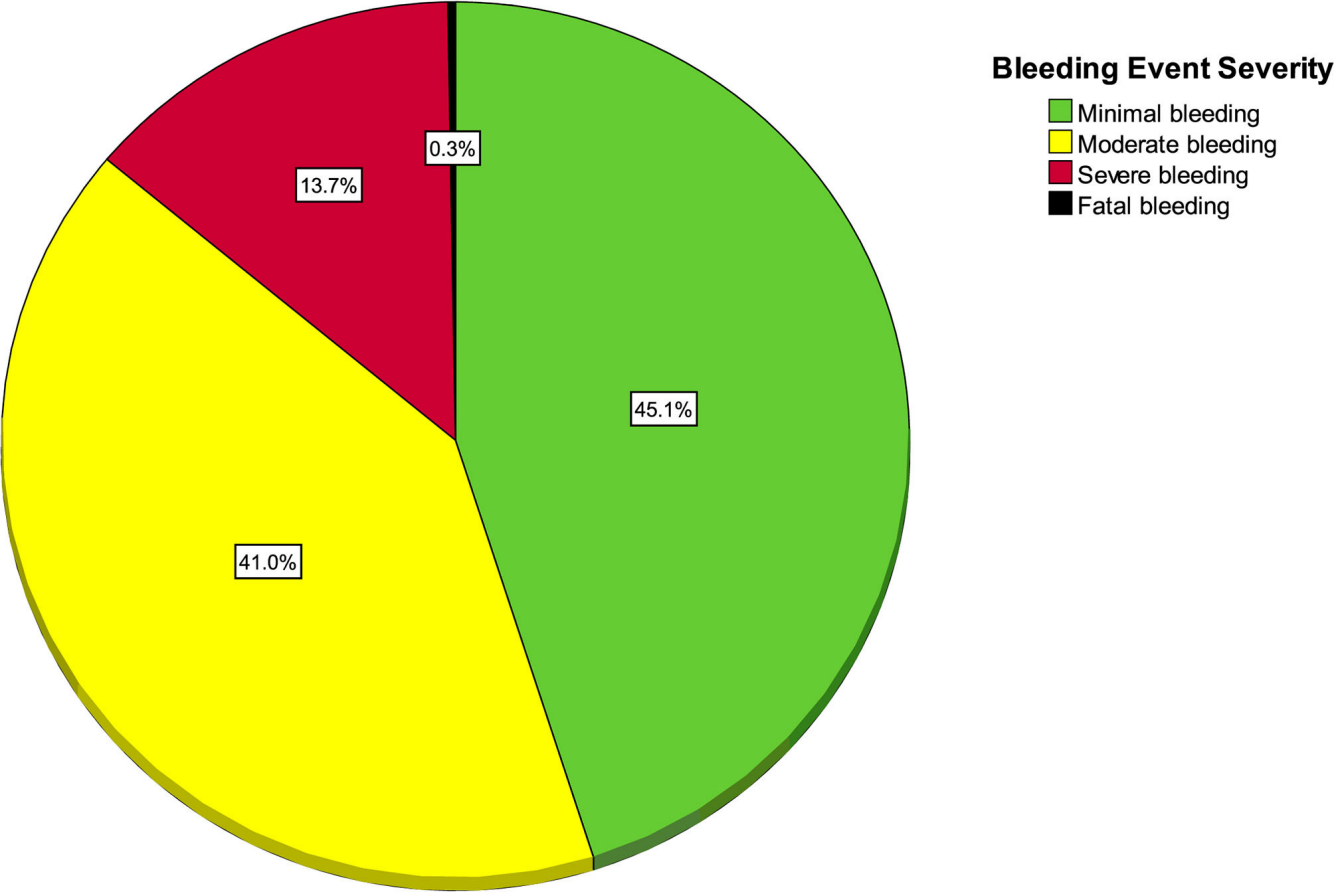
Pie chart of bleeding severity of each event (*n* = 366 bleeding events).

As seen in Figure 2, out of 366 bleeding events, there were 165 (45.1%, 95% CI 40.0 to 50.0) minimal bleeding events, 150 (41.0%, 95% CI 36.1 to 46.1) moderate, 50 (13.7%, 95% CI 10.5 to 17.6%) severe, and 1 (0.2%, 95% CI 0.1 to 1.5) fatal event in a trauma patient. The only fatal bleeding event was a patient with intracranial hemorrhage.

The median number of bleeding episodes per patient was 2 (IQR 1; 3). One hundred and fifty-nine (43%) children had only one episode, 88 (24%) had two episodes, 47 (13%) had three episodes, and 72 (20%) had more than three episodes. There was no statistical association between the severity of bleeding and the number of bleeding events (*p* = 0.96).

Of note, the patient on ECMO did not have a bleeding event.

### Description of Individual Bleeding Criteria

As shown in Table 2, the most frequent sources of minimal bleeding were quantifiable bleeding (53.3%) and bloody dressings (33.9%).

**Table 2:** Criteria for minimal (*n* = 165) and severe bleeding (*n* = 51).

**Minimal bleeding criteria**	
Streaks of blood in ETT or during suctioning only	8 (4.8%)
Streaks of blood in nasogastric tube	4 (2.4%)
Macroscopic hematuria, or ≤1+ RBCs present on urine dipstick if available	7 (4.2%)
Subcutaneous bleeding (including hematoma and petechiae) <5 cm (2 inches) in diameter	3 (1.8%)
Quantifiable bleeding < 1 ml/kg/h (e.g., chest tube, drain)	88 (53.3%)
Bloody dressings required to be changed no less than each 6 h, or weighing no >1 ml/kg/h if weighed, due to slow saturation	56 (33.9%)
**Severe bleeding criteria**	
Bleeding that leads to at least one organ dysfunction, using PELOD-2 score criteria of organ dysfunction, within 24 h of the previous assessment (if there is no previous assessment, the baseline results are presumed to be normal)	29 (56.9%)
Bleeding that leads to hemodynamic instability, defined as an increase in heart rate by >20% from baseline or a decrease in blood pressure by >20% from baseline	15 (29.4%)
Bleeding leading to a drop in hemoglobin >20% within 24 h	13 (25.5%)
Quantifiable bleeding ≥5 ml/kg/h for ≥1 h (e.g., chest tube, drain)	14 (27.5%)
Intraspinal bleeding leading to loss of neurologic function below the lesion, non-traumatic intra-articular bleeding leading to decreased range of movement, or intraocular bleeding leading to impaired vision	3 (5.9%)

*ETT, endotracheal tube; RBC, red blood cell; PELOD, Pediatric Logistic Organ Dysfunction*.

The most frequent criteria that led to severe bleeding was an increase in org an dysfunction (56.9%, Table 2). Hemodynamic instability, 20% drop in hemoglobin, and quantifiable bleeding >5 ml/kg/h were each reported to contribute to severe bleeding in a quarter of the events.

### Interventions Associated With Bleeding

Forty-three patients (11.7% of bleeding patients, 4.8% of all patients) received blood product transfusions to treat bleeding: 24 (6.6% of bleeding patients, 2.7% of all patients) received red blood cells, 19 (5.2% of bleeding patients, 2.1% of all patients) plasma, 15 (4.1% of bleeding patients, 1.6% of all patients) platelets, and 15 (4.1% of bleeding patients, 1.6% of all patients) cryoprecipitate. The severity of bleeding was associated with higher doses of red blood cells (*p* = 0.004), platelets (*p* = 0.04), and cryoprecipitate (*p* = 0.03), but not plasma (*p* = 0.47).

Thirteen patients (3.6%) required surgical interventions (including four neurosurgical interventions and four surgical chest or abdominal explorations) or procedure (one bronchoalveolar lavage and one chest tube insertion) to stop bleeding. The severity of bleeding was associated with the need for surgical interventions or procedures (*p* < 0.001). In addition, two patients received vitamin K and one patient received protamine. No patients required prothrombin concentrates, recombinant factor V, or for anticoagulation to be discontinued.

### Risk Factors for Severity of Bleeding

Among medical patients, the severity of bleeding was independently associated with severity at admission and admission diagnosis of gastrointestinal bleeding (the regression models are available in the Supplementary Material). Admission diagnoses of seizure, encephalopathy, and respiratory distress independently decreased the risk of severe bleeding.

Among surgical patients, the severity of bleeding was independently associated with severity at admission, admission diagnosis of trauma or infection, emergent surgery, and length of surgery, whereas, age and weight were not. Surgical categories independently associated with severity of bleeding were intracranial neurosurgery, congenital cardiac surgery, thoracic surgery, spinal surgery, ENT, post solid-organ transplantation, and plastic surgery. Post-surgical severity of bleeding was also independently associated with intraoperative need for vascular repair, use of plasma, and tranexamic acid, whereas, the use of cell-saver, intraoperative use of cryoprecipitate, and platelets were not.

Among patients who experienced bleeding, neither underlying coagulopathy nor iatrogenically altered hemostasis appeared to be significantly associated with an increased risk of bleeding, seen in Table 3.

**Table 3:** Underlying conditions potentially associated with risk of bleeding, evaluated on the day of bleeding.

**Variables**	**Minimal bleeding *n* = 165**	**Moderate bleeding *n* = 150**	**Severe bleeding** ^*^ ***n* = 51**	***P*-value**
Anticoagulation	8 (4.8%)	7 (4.7%)	2 (3.9%)	0.96
Anti-platelet therapy	3 (1.8%)	5 (3.3%)	0 (0%)	0.33
Chemotherapy	3 (1.8%)	3 (2.0%)	0 (0%)	0.96
Underlying coagulopathy^**^	7 (4.2%)	3 (2.0%)	3 (5.9%)	0.32

* *Including 1 case of fatal bleeding (intracranial hemorrhage)*.

** *Including thrombocytopenia, abnormal coagulation tests, and known factor deficiencies (e.g., Von Willebrand, hemophilia)*.

### Outcomes

**Figure 3:**
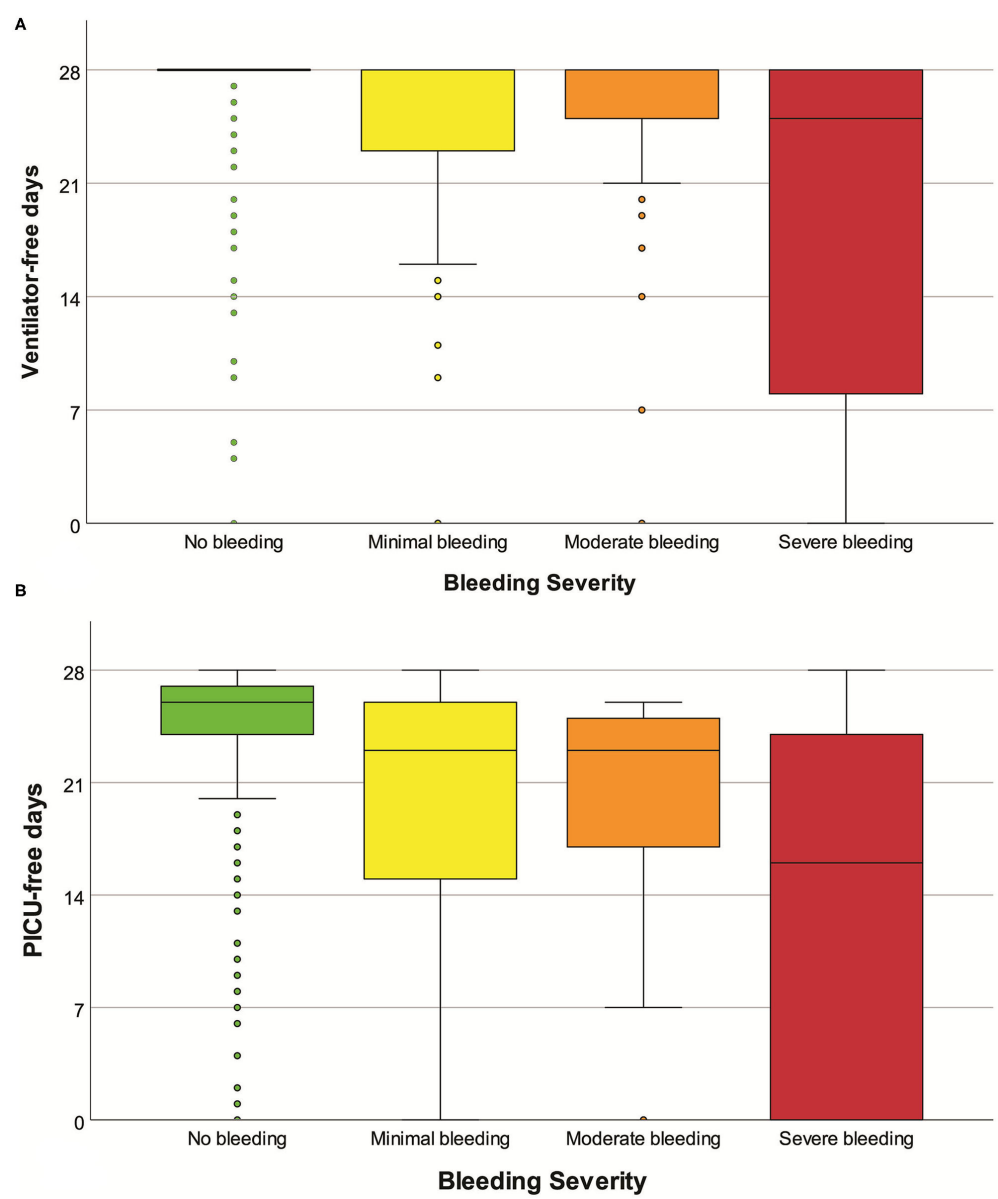
Boxplot of ventilator-free days **(A)** and PICU-free days **(B)**, according to the severity of bleeding. Mild outliers (<1.5 IQR) and extreme outliers (≥1.5 IQR) are marked with circles and asterisks, respectively.

At a univariate level, the severity of bleeding was significantly associated with various clinical outcomes (Table 4 and Figure 3).

**Table 4:** Clinical outcomes of the entire cohort (*n* = 902).

**Variables**	**Highest severity of bleeding**				***P*-value**
	**No**	**Minimal**	**Moderate**	**Severe**	
	**bleeding**	**bleeding**	**bleeding**	**bleeding** ^*^	
	**(*n* = 744)**	**(*n* = 71)**	**(*n* = 52)**	**(*n* = 35)**	
Days of mechanical ventilation	0 (0; 0)	0 (0; 5)	0 (0; 3)	2 (0; 9)	<0.001
Ventilator-free days	28 (28; 28)	28 (23; 28)	28 (25; 28)	25 (8; 28)	<0.001
PICU length of stay	2 (1; 4)	5 (2; 13)	5 (3; 11)	7 (3; 15)	<0.001
PICU-free days	26 (24; 27)	23 (15; 26)	23 (17; 25)	16 (0; 24)	<0.001
Mortality	14 (1.9%)	5 (7.0%)	1 (1.9%)	7 (20.0%)	<0.001

* *Including 1 case of fatal bleeding (intracranial hemorrhage)*.

*PICU, pediatric intensive care unit*.

Adjusting for weight, gender, severity at admission, admission diagnosis, and duration of surgery, bleeding severity was independently associated with fewer ventilator-free days (adjusted ventilator-free days for each bleeding category −2.5, 95% CI −3.2; −1.8, *p* < 0.001) and fewer PICU-free days (*p* < 0.001). Adjusting for the same variables, bleeding severity was independently associated with an increased risk of mortality (adjusted odds ratio for each bleeding category 2.4, 95% CI 1.5; 3.7, *p* < 0.001). Results from the regression models are available in the Supplementary Material.

## Discussion

Our study, which sought to describe the epidemiology and clinical burden of bleeding in the critically ill pediatric population, suggested that bleeding occurs in ~18% of patients, and that disease severity at admission was the most significant predictor of severity of bleeding in both medical and surgical patients. In medical patients, an admission diagnosis of gastrointestinal bleeding was the most significant predictor of bleeding severity, while for surgical patients, bleeding severity was predicted by trauma or infection admission diagnosis, as well as total length of surgery. Furthermore, bleeding severity was independently associated with fewer ventilator-free days, fewer PICU-free days, and increased mortality risk. This study also provides further validation of BASIC bleeding definition, indicating association between degree of bleeding severity and clinical outcomes.

Due to the heterogeneity of the concept of “clinically significant bleeding,” the epidemiology and outcomes of bleeding are difficult to accurately quantify. Adult studies have reported bleeding incidence at 30% in BMT patients, while some studies report bleeding incidence as high as 90% in critically ill populations, with subsequent longer ICU stays, albeit variable survival (4, 12, 13). Pinto et al. (2) showed that in critically ill adolescents, this incidence was 30%, and when studying specifically critically ill children, White et al. (3) estimated the bleeding incidence to be 37%, with clinically significant bleeds only occurring in 9% of patients. Greenway et al. (14) identified a similar incidence of clinically relevant bleeds in critically ill children; 10% using the ISTH definition. It should be noted that White et al. excluded patients bleeding at admission, which eliminated a large number of surgical patients from the bleeding cohort, and was likely contributory to the subsequently decreased risk of bleeding associated with surgery when compared to our cohort. It should also be noted that both of these studies defined clinical relevant bleeding using the International Society on Thrombosis and Hemostasis (ISTH) categories of bleeding, which were designed to standardize outcomes in clinical trials of anticoagulation (15, 16). These definitions fail to account for differences in pediatric physiology, and are highly dependent on subjective assessments as the definition links the decision to transfuse blood products to the severity of bleeding. As such, the ISTH definition may not completely capture all nuances of bleeding in a critical care setting or may overemphasize the severity of a bleed.

Our study showed that 12% of bleeding pediatric patients received blood products, and that severity of bleeding was associated with higher doses of all blood products except plasma. In critically ill adults, transfusion rates are significantly higher, with 37% of patients receiving a transfusion during their ICU stay, and over two thirds of those patients requiring multiple transfusions (16). However, the need for transfusion was included in their definition of severe bleeding. Transfused patients had more ICU days, more severe organ failure, and higher mortality than non-transfused patients (17). Regarding plasma transfusions, a multicenter trial of 13,192 critically ill children identified that 1.5% received plasma to treat bleeding, compared to 2.1% in this current cohort (18).

Hemostatic transfusions are frequently administered to critically ill patients to prevent or stop bleeding. In critically ill adult patients with hypoproliferative thrombocytopenia, platelet transfusions occurred in over 44% of patients, but were of limited benefit, with poor post-transfusion improvement increments (19). Prophylactic transfusions are also extremely common in pediatric patients with oncologic disorders. Nellis et at. demonstrated that 43% of critically ill pediatric patients who receive a platelet transfusion have an underlying oncologic diagnosis. In these patients, 71% of the transfusions were administered prophylactically. Patients with oncologic diagnoses demonstrated impaired increment response to platelets, and while they received more transfusions overall, their total platelet dose over the admission was equivalent to their non-oncological cohorts, and mortality was not significantly different (20). This is consistent with our findings, that while a moderate number of bleeding patients had an oncologic diagnosis, this diagnosis did not independently increase the risk of a severe bleeding event. When looking at a general population of critically ill children, a large international study of 16,934 pediatric patients showed that 3.3% received platelets to treat bleeding, compared to 1.6% in our study (21).

We furthermore, showed that 3.6% of patients required surgical interventions, and that severity of bleeding was also associated with the need for surgical interventions. Previous studies have shown that some of the most frequent triggers for transfusion are preoperative, to correct laboratory values prior to an invasive procedure in the absence of clinical bleeding. Karam et al. (18) previously demonstrated that 34% of critically ill patients received plasma prior to a planned procedure.

Our study had several limitations. First, in the critically ill population, the nature of some critical interventions performed while resuscitating patients may have increased the odds of bleeding, in which case bleeding would be a marker of severity, instead of being independently associated with worse outcome. Therefore, we might have overestimated the real association between bleeding severity and outcomes. For example, bleeding from an endotracheal tube might be a consequence of the need for intubation and mechanical ventilation, which is itself a risk factor for worse outcome. Second, data was not collected on weekends and holidays, due to staffing restrictions, which might have led to a selection bias as elective surgeries are frequently done during the week. Third, while our center does encompass trauma, ECMO, cancer, BMT and solid organ transplants, our study was single center and as such may not be fully generalizable to other units. Finally, bedside nursing assessments and documentation likely led to variation in data capture, for example when different nurses would assess the same patient's clinical bleeding as less or more severe, or when documentation of blood loss was charted all at once, instead of hourly, making rate calculations unreliable.

## Conclusion

In conclusion, bleeding in the critically ill pediatric population is frequent and represents a significant source of morbidity and mortality. Further, studies will be required to elaborate the importance of laboratory values and clinical findings in the decision of the clinician to intervene to address bleeding events, as well as the impact blood product administration has on outcomes.

## Data Availability Statement

The raw data supporting the conclusions of this article will be made available by the authors, without undue reservation.

## Ethics Statement

The studies involving human participants were reviewed and approved by Virginia Commonwealth University Institutional Review Board (HM20016435). Written informed consent from the participants' legal guardian/next of kin was not required to participate in this study in accordance with the national legislation and the institutional requirements.

## Author Contributions

JS wrote the research project, analyzed data, and wrote the manuscript. OK wrote the research project, analyzed the data, and reviewed the manuscript. MN wrote the research project and reviewed the manuscript. JS and OK had full access to the data and take responsibility for its integrity. All authors contributed to the article and approved the submitted version.

## Conflict of Interest

The authors declare that the research was conducted in the absence of any commercial or financial relationships that could be construed as a potential conflict of interest.

## Publisher's Note

All claims expressed in this article are solely those of the authors and do not necessarily represent those of their affiliated organizations, or those of the publisher, the editors and the reviewers. Any product that may be evaluated in this article, or claim that may be made by its manufacturer, is not guaranteed or endorsed by the publisher.
