# Supplementary material for: Epidemiology of Bleeding in Critically Ill Children
Source: Front Pediatr. 2021 Aug 4;9:699991. doi: 10.3389/fped.2021.699991 (PMC8371326; doi:10.3389/fped.2021.699991)
Supplement: Supplementary file 1 [file Data_Sheet_1.PDF]

Epidemiology of Bleeding in Critically Ill Children

Jake Sequeira, MD; Marianne E Nellis, MD, MS; Oliver Karam, MD, PhD

Supplemental tables

Table S1: Ordinal regression model for bleeding severity in medical patients

	Estimate	95% Confidence Interval Lower Bound	Upper Bound	p value
Age	0.004	-0.001	0.01	0.113
Weight	-0.004	-0.02	0.012	0.631
PIM-2	0.007	0.0008	0.014	**0.047**
Hypovolemic shock	-0.187	-2.071	1.697	0.846
Severe sepsis and septic shock	-0.756	-1.728	0.216	0.127
GI bleeding	2.066	0.3	3.832	**0.022**
Cardiogenic shock	-1.086	-2.98	0.808	0.261
Burn	-2.164	-4.346	0.019	0.052
Traumatic Brain Injury	0.988	-0.116	2.093	0.079
Trauma	-0.551	-1.386	0.284	0.196
Seizures	-2.387	-3.609	-1.165	**<0.001**
Encephalopathy	-2.884	-4.495	-1.272	**<0.001**
Renal failure	-1.122	-2.856	0.612	0.205
Respiratory distress (infectious)	-2.76	-3.846	-1.673	**<0.001**
Respiratory distress (non-infectious)	-2.543	-3.619	-1.467	**<0.001**
Oncology	0.081	-0.712	0.873	0.842
Endocrine (including DKA)	-19.63	-4435.838	4396.578	0.993
Infection	-19.586	-8770.086	8730.913	0.996

Table S2: Ordinal regression model for bleeding severity in surgical patients

	Estimate	95% Confidence Interval Lower Bound	Upper Bound	p value
Age	0.003	-0.002	0.009	0.194
Weight	0.001	-0.005	0.006	0.774
PIM-2	0.021	0.011	0.031	**<0.001**
Length of surgery	0.196	0.042	0.35	**0.013**
Cellsaver	-0.001	-0.003	0.001	0.176
Tranexamic acid during surgery	0.034	0.014	0.054	**0.001**
Platelets during surgery	0.126	-0.045	0.296	0.148
Plasma during surgery	0.047	0.002	0.097	**0.049**
Cryoprecipitate during surgery	-2.384	-5.687	0.92	0.157
Burn	0.908	-3.23	5.046	0.667
Traumatic brain Injury	1.893	-2.427	6.213	0.39
Trauma	3.481	1.877	5.085	**<0.001**
Cardiac surgery (bypass)	0.939	-1.427	3.305	0.436
Emergent surgery	2.799	0.869	4.73	**0.004**
Respiratory distress (non-infectious)	1.861	-0.813	4.536	0.173
Oncology	-19.22	-9211.431	9172.991	0.997
Infection	5.2	2.725	7.675	**<0.001**
Neuro (other)	-18.082	-9441.26	9405.096	0.997
Intracranial neurosurgery	2.061	0.798	3.325	**0.001**
Cardiac congenital surgery	2.097	0.09	4.284	**0.048**
Thoracic surgery	4.312	2.656	5.968	**<0.001**
Spinal surgery	3.834	2.059	5.608	**<0.001**
Cranial vault surgery	0.625	-1.475	2.725	0.56
ENT	2.823	1.259	4.388	**<0.001**
Transplant	2.105	0.303	3.907	**0.022**
Plastic	2.232	0.633	3.831	**0.006**
Surgery requiring vascular repair	1.338	0.42	2.255	**0.004**
Surgery requiring prolonged hemostasis	0.459	-0.706	1.624	0.44

Table S3: Linear regression model for ventilation-free days

	B	95% Confidence Interval for B<br>Lower Bound	 Upper Bound	p value
Bleeding severity	-2.549	-3.201	-1.896	**<0.001**
Weight	0.009	-0.001	0.02	0.091
Gender	-0.902	-2.048	0.244	0.122
PIM-2	0.012	-0.007	0.03	0.218
Admission Diagnosis	-0.019	-0.061	0.024	0.38
Length of surgery	0.25	0.014	0.487	**0.038**
Constant	*28.678*	*26.704*	*30.652*	*<0.001*

Table S4: Linear regression model for PICU-free days

		95% Confidence Interval for B		
	B	Lower Bound	Upper Bound	p value
Bleeding severity	-2.825	-3.642	-2.007	**<0.001**
Weight	0.007	-0.006	0.02	0.309
Gender	-1.728	-3.164	-0.293	**0.019**
PIM-2	0.005	-0.018	0.028	0.671
Admission diagnosis	-0.071	-0.124	-0.018	**0.009**
Length of surgery	0.153	-0.143	0.449	0.311
Constant	*27.886*	*25.414*	*30.358*	*<0.001*

Table S5: Logistic regression model for mortality

	Exp(B)	95% C.I. for EXP(B)		p value
		Lower	Upper	
Bleeding Severity	2.38	1.511	3.689	**<0.001**
Weight	0.978	0.949	1.008	0.146
PIM-2	1.036	1.022	1.05	**<0.001**
Male	1.371	0.43	4.372	0.594
Severe sepsis and septic shock	1.958	0.12	31.933	0.637
Cardiogenic shock	16.136	0.762	341.526	0.074
Traumatic brain injury	0.667	0.014	32.916	0.839
Traumatic brain injury	2.021	0.156	26.126	0.59
Encephalopathy	7.03	0.588	83.996	0.123
Renal Failure	8.707	0.262	289.047	0.226
Respiratory failure (non-infectious)	1.605	0.105	24.597	0.734
Oncology	1.751	0.119	25.655	0.683
Infection	3.88	0.157	95.839	0.407
Length of surgery	0.144	0.024	0.85	**0.032**
Constant	*0.01*			*<0.001*
